# Prediction model for hyperprogressive disease in patients with advanced solid tumors received immune-checkpoint inhibitors: a pan-cancer study

**DOI:** 10.1186/s12935-023-03070-x

**Published:** 2023-09-30

**Authors:** Yaping Long, Wenyu Yang, Yibing Bai, Haitao Tao, Fan Zhang, Lijie Wang, Bo Yang, Di Huang, Xiao Han, Yi Hu

**Affiliations:** 1 School of Medicine, Nankai University, 94 Weijin Road, Nankai, Tianjin, China; 2 PLA General Hospital, 28 Fuxing Road, Haidian District, Beijing, 100853 People’s Republic of China; 3 Department of Medical Oncology, Department of Medical Oncology, Senior Department of Oncology, The Fifth Medical Center of PLA General Hospital, 8 Dongdajie Road, Fengtai District, Beijing, 100071 China

**Keywords:** Hyperprogressive disease, Immune checkpoint inhibitor, Anemia, Predictive model, Pan-cancer analysis, ICI response

## Abstract

**Background:**

Hyper progressive disease (HPD) describes the phenomenon that patients can’t benefit from immunotherapy but cause rapid tumor progression. HPD is a particular phenomenon in immunotherapy but lacks prediction methods. Our study aims to screen the factors that may forecast HPD and provide a predictive model for risky stratifying.

**Methods:**

We retrospectively reviewed advanced-stage tumor patients who received immune checkpoint inhibitors (ICI) in the General PLA Hospital. Subsequently, we calculated the tumor growth kinetics ratio (TGKr) and identified typical HPD patients. Differences analysis of clinical characteristics was performed, and a predictive binary classification model was constructed.

**Results:**

867 patients with complete image information were screened from more than 3000 patients who received ICI between January 2015 and January 2020. Among them, 36 patients were identified as HPD for TGKr > 2. After the propensity score matched, confounding factors were limited. Survival analysis revealed that the clinical outcome of HPD patients was significantly worse than non-HPD patients. Besides, we found that Body Mass Index (BMI), anemia, lymph node metastasis in non-draining areas, pancreatic metastasis, and whether combined with anti-angiogenesis or chemotherapy therapy were closely connected with the HPD incidence. Based on these risk factors, we constructed a visualised predicted nomogram model, and the Area Under Curve (AUC) is 0.850 in the train dataset, whereas 0.812 in the test dataset.

**Conclusion:**

We carried out a retrospective study for HPD based on real-world patients and constructed a clinically feasible and practical model for predicting HPD incidence, which could help oncologists to stratify risky patients and select treatment strategies.

**Supplementary Information:**

The online version contains supplementary material available at 10.1186/s12935-023-03070-x.

## Introduction

Hyper progressive disease (HPD) was identified as patients deteriorated rapidly after receiving immune checkpoint inhibitor (ICI) treatment, associated with a poor prognosis in multiple solid tumors [1, 2]. Ambiguous mechanisms and lacking an efficacious predictive approach led to a therapeutic dilemma in clinical ICI strategies [3]. Our study aims to provide a predictive model based on patients’ clinical characteristics and lab tests for the incidence of HPD, which fits into clinical use.

The incidence of HPD is not rare, which is reported to be 4% to 29% in the previous study [4]. Also, it can occur in most malignant tumors, regardless of the specific ICI drug type. Despite the objective diagnostic criteria for HPD remaining controversial [5, 6], there are currently recognized diagnostic standards, including (1) Time to tumor progression less than two months after patients receive ICI treatment. (2) More than a 50% increase in tumor volume compared to the baseline. (3) The tumor growth kinetics ratio (TGKr) is the most widespread method to evaluate HPD, and TGKr > 2 is considered the standard cutoff for disease occurrence [7].

The molecular mechanism underlying HPD incidence needs to be better defined [8]. From various studies, there are some hypotheses for the phenomenon. First, blockade of Programmed Death-1(PD-1) or Programmed cell death 1 ligand 1 (PD-L1) will enhance the function of Treg cell [9], leading to an immunosuppressive tumour microenvironment [10]. A compensatory increase in checkpoints after immunotherapy will cause T-cell inactivation. Besides, ICI treatment may polarize the immune cell subset to an immunosuppressive phenotype, capable of secreting immunosuppressive cytokines and reducing effector T-cell proliferation. Moreover, the Fc receptor of tumor-associated macrophage reprogramming accelerates immune escape and tumor growth [11]. The PD-1/PD-L1 inhibitor may activate oncogenic signalling pathways and promote tumor cell proliferation [12–14]. Finally, there may be an immune and metabolic pathway intersection [15].

Predicting the incidence of HPD is essential in avoiding short survival and quality-of-life deterioration. Previous studies found that advanced age [1], number of metastases [16], a difference of T-cell phenotype in blood [17], and Mouse Double Minute 2 (MDM2) amplifications [4, 18], Kirsten Rat Sarcoma Viral Oncogene Homolog (KRAS), and Serine/Threonine Kinase 11 (STK11) mutation [19] are associated with HPD. Contrarily, HPD is unrelated to the tumor burden, therapeutic regime, and PD-L1 expression status, which is closely linked to the therapeutic effect of ICI in the traditional view.

Research on HPD has become a hot spot in oncology and immunology. However, rigorous clinical trials aimed at screening predictors for HPD are insufficient, or the practical value of factors needs to be met for clinicians. Therefore, our team reviewed the patients who received ICI treatment, identified the typical HPD group, and constructed a well-prognostic model with easily accessible clinical indicators to provide a reference for the clinical judgment of oncologists.

## Materials and methods

### Patients selection and study design

**Fig. 1:**
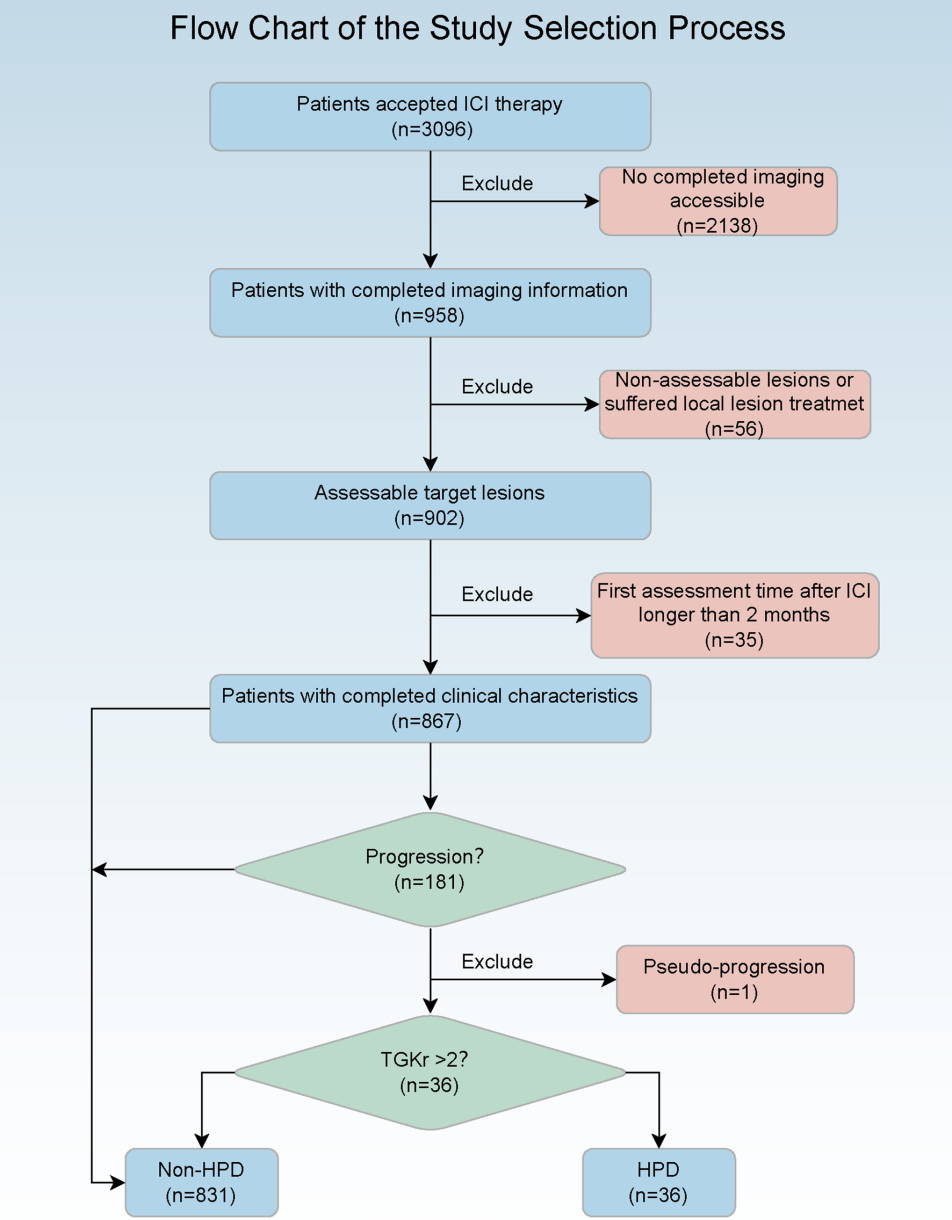
Flow diagram of the patients’ screening process

This was a retrospective observational single-center study. Between January 2015 and January 2020, 3096 patients who received anti-PD-1 therapy with complete follow-up information in the Chinese PLA general hospital were enrolled in this retrospective study. Inclusion and exclusion criteria were: (a) The pathological diagnosis of patients identified as carcinoma by biopsy or surgical resection. (b) Completed imaging data (including pre-baseline, baseline, and first assessment after ICI therapy imaging, at least) to calculate TGKr and identify HPD incidence. (c) Assessable target lesions could be measured by Computer Tomography (CT) or Magnetic Resonance Imaging (MRI) scan based on Response Evaluation Criteria In Solid Tumors (RECIST) 1.1. (d) Lesions that suffered local treatment were excluded. (e) First assessment time after ICI is shorter or equal to 2 months, and the time of the pre-baseline is less than 3 months. The study design was shown in Fig. 1.

### Tumor assessments and HPD Definition

Imagining data were judged by two independent senior oncologists, and recorded the sum of the tumor diameter of the target lesions (D) to assess tumor growth at three-time points (T): the time of the pre-baseline (Tpre), baseline (T0), and first assessment imaging, respectively, after receiving immunotherapy (Tpost). The valid time of tumor evaluation time point needs to meet Tpre < 3 months, T0 < 2 weeks, and Tpost < 2 months. The target lesions were reassessed for each patient at the tumor evaluation timepoint using RECIST 1.1.

**Fig. 2:**
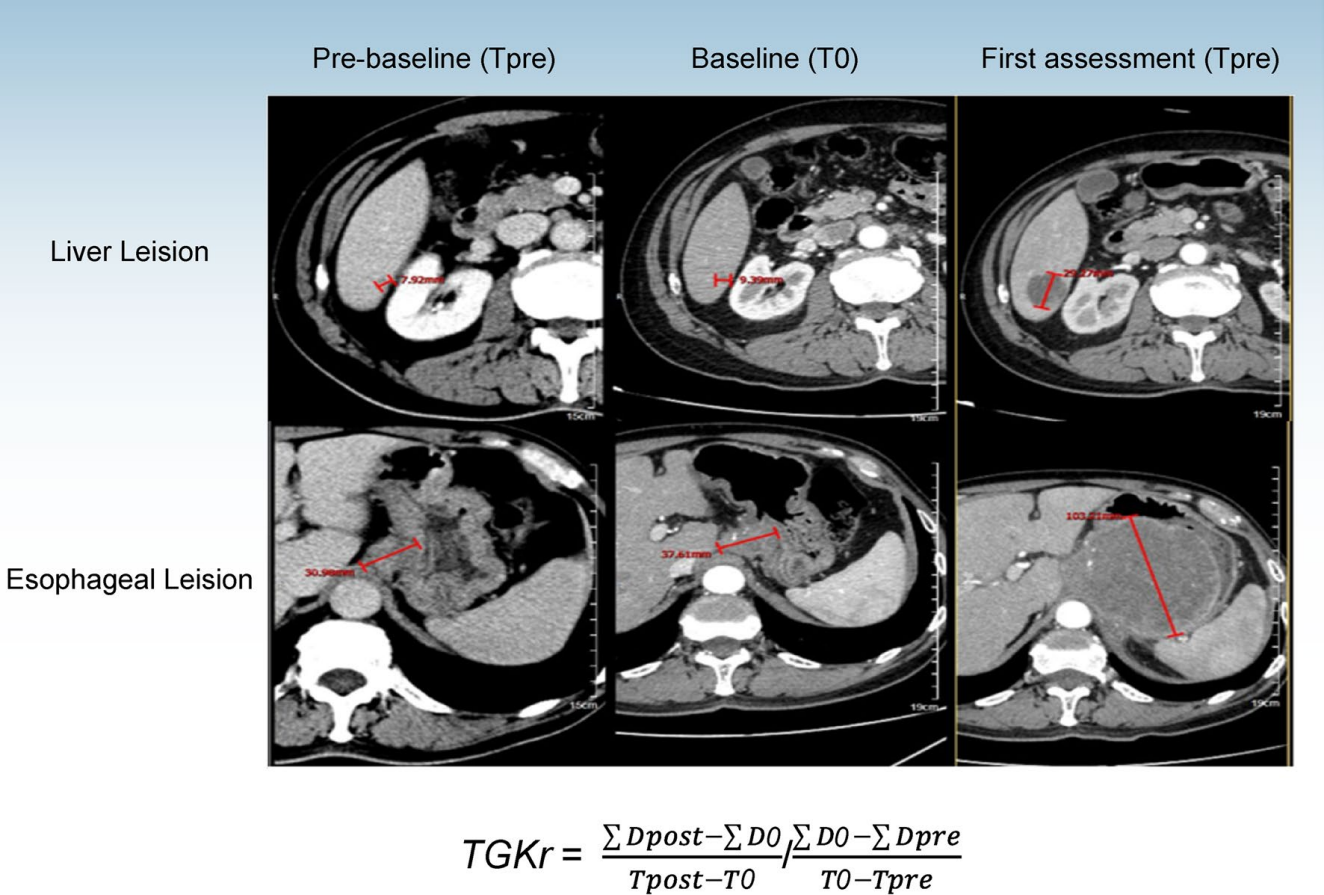
Thoracic and abdominal CT review results showed accelerated tumor progression. **A**. Pre-baseline assessment: the cross-sectional diameter of the right lobe of the liver was 4.62 mm, and the cross-section diameter of the lesion with a slight density of the esophageal was 21.17 mm; **B**. Baseline assessment: the cross-sectional diameter of the right lobe of the liver was 8.73 mm, and the cross-section diameter of the lesion with a slight density of the esophageal space was 28.17 mm; **C**. First assessment after ICI evaluation: the cross-sectional diameter of the right lobe of the liver was 42.18 mm, and the cross-section diameter of the lesion with a slight density of the esophageal space was 101.02 mm

Tumor growth kinetics ratio (TGKr) model was used to assess antitumor efficacy. The formula is TGKr = $\frac{\sum Dpost-\sum D0}{Tpost-T0}$/$\frac{\sum Do-\sum Dpre}{T0-T\mathrm{pre}}$, and patients with TGKr > 2 was identified as HPD [17, 20, 21]. Besides, pseudo-progression after ICI therapy was excluded [22]. Typical imaging of HPD patients were shown in Fig. 2.

### Differential analysis between HPD and non-HPD groups

Dependent on TGKr methods, patients were classified into HPD and non-HPD groups. Kaplan–Meier curve analysis with a log-rank test was conducted to perform the differences in overall survival (OS) and progression-free survival (PFS) between the two groups. Subsequently, χ^2^ and Fisher’s exact tests were performed to identify associations between clinical characteristics and the HPD groups. T-test or Wilcoxon test was applied to analyze the connection between lab tests and the HPD groups. Propensity score matching (PSM) was adopted to balance the distribution differences with a matching ratio of 1:6. After PSM, the survival analysis was performed.

### Construction of the predictive nomogram

Patients were randomly divided into train and test datasets at the ratio of 7:3. Based on the significantly different clinical characteristics and lab tests, we performed multivariate logistics analysis and constructed a predictive nomogram model. ROC and precision-recall curve was used to assess the predictive ability of the model in both train and test databases, and the Hosmer-Lemeshow test was conducted to perform the consistency between the actual incidence of HPD and the predicted incidence of HPD.

### Statistical analysis

Statistical analysis was performed in R Version 4.1.0, SPSS 27.0 and GraphPad prism 9. The clinical outcomes between the two groups were assessed using the Kaplan–Meier curve analysis and the Log-rank test. T test or Mann–Whitney’s test for continuous variables, the χ ^2^ or Fisher’s exact tests for categorical data. The PSM was conducted by R package “MatchIt”. The nomogram was performed by R package “rms” and examined by R package “pROC” and “modEvA”. The statistically significant difference was determined by “p-value < 0.05”.

## Results

After the screening, 867 patients who received ICI therapy with complete clinical information were enrolled in our study. According to TGKr, 36 patients were identified as HPD, whereas 831 patients were in the non-HPD group. The distribution of clinical characteristics divided by HPD groups were listed in Table 1. The majority of patients were male (629/867, 72.5%). Incidence of HPD varied statistically significantly among pathological types (p = 0.008). Patients with pancreatic metastasis (p < 0.001) and non- draining lymph node metastasis (p < 0.001) had more intendency to undergo HPD. The incidences of HPD decreased in patients combined chemotherapy (p = 0.001), antiangiotherapeutics (p = 0.005) or other therapy (p < 0.001) while patients treated with ICIs. Patients in HPD groups has lower BMI (p < 0.001) and weight (p < 0.001) compared with non-HPD groups.

**Table 1:** Clinical characteristics of patients accepted ICI therapy

Characteristic	Non-HPD	HPD	p value	Method
N	831	36		
Gender, N (%)			0.032	Chisq.test
Female	222 (26.71%)	16 (1.93%)		
Male	609 (73.29%)	20 (2.41%)		
Pathology Type, N (%)			0.008	Chisq.test
Adenocarcinoma	411 (49.46%)	18 (2.17%)		
Sarcoma	10 (1.2%)	3 (0.36%)		
Small Cell Carcinoma	145 (17.45%)	4 (0.48%)		
Squamous	188 (22.62%)	6 (0.72%)		
Others	77 (9.27%)	5 (0.6%)		
Differentiation, N (%)			0.349	Chisq.test
Low Differentiation	442 (53.19%)	14 (1.68%)		
Low To Median Differentiation	78 (9.39%)	5 (0.6%)		
Median Differentiation	131 (15.76%)	7 (0.84%)		
Median To High Differentiation	14 (1.68%)	2 (0.24%)		
High Differentiation	6 (0.72%)	0 (0)		
Unknown	160 (19.25%)	8 (0.96%)		
Ki-67, N (%)			0.989	Chisq.test
0–25%	51 (6.14%)	2 (0.24%)		
26–50%	71 (8.54%)	4 (0.48%)		
51–75%	91 (10.95%)	4 (0.48%)		
76–100%	93 (11.19%)	4 (0.48%)		
Unknown	525 (63.18%)	22 (2.65%)		
TNM Stage, N (%)			0.264	Chisq.test
III	76 (9.15%)	0 (0)		
IV	725 (87.24%)	36 (4.33%)		
Unknown	22 (2.65%)	0 (0)		
Smoking, N (%)			0.589	Fisher.test
Current	146 (17.57%)	8 (0.96%)		
Ever	288 (34.66%)	10 (1.2%)		
Never	381 (45.85%)	17 (2.05%)		
Unknown	16 (1.93%)	1 (0.12%)		
Drinking, N (%)			0.702	Fisher.test
Current	202 (24.31%)	10 (1.2%)		
Ever	155 (18.65%)	5 (0.6%)		
Never	459 (55.23%)	20 (2.41%)		
Unknown	15 (1.81%)	1 (0.12%)		
Family History of Tumor, N (%)			0.525	Chisq.test
Yes	226 (27.2%)	8 (0.96%)		
No	456 (54.87%)	19 (2.29%)		
Unknown	149 (17.93%)	9 (1.08%)		
KPS score, N (%)			0.256	Chisq.test
10–20 point	30 (3.61%)	2 (0.24%)		
30–40 point	5 (0.6%)	0 (0)		
70–80 point	125 (15.04%)	10 (1.2%)		
90–100 point	661 (79.54%)	24 (2.89%)		
Unknown	10 (1.2%)	0 (0)		
Combined Chemotherapy, N (%)			0.001	Chisq.test
No	343 (41.28%)	25 (3.01%)		
Yes	488 (58.72%)	11 (1.32%)		
Combined Antiangio, N (%)			0.005	Chisq.test
No	594 (71.48%)	34 (4.09%)		
Yes	237 (28.52%)	2 (0.24%)		
Combined Target Therapy,N (%)			1.000	Fisher.test
No	794 (95.55%)	35 (4.21%)		
Yes	37 (4.45%)	1 (0.12%)		
Combined Radiotherapy, N (%)			0.421	Fisher.test
No	790 (95.07%)	33 (3.97%)		
Yes	41 (4.93%)	3 (0.36%)		
Combined Other Therapy, N (%)			< 0.001	Chisq.test
No	241 (29%)	22 (2.65%)		
Yes	590 (71%)	14 (1.68%)		
Count of Metastasis Lesion, N (%)			0.386	Chisq.test
0	62 (7.46%)	1 (0.12%)		
1	77 (9.27%)	4 (0.48%)		
2	71 (8.54%)	2 (0.24%)		
3	72 (8.66%)	1 (0.12%)		
4	57 (6.86%)	1 (0.12%)		
More than 4	492 (59.21%)	27 (3.25%)		
Lung Metastasis, N (%)			0.645	Chisq.test
No	371 (44.65%)	18 (2.17%)		
Yes	460 (55.35%)	18 (2.17%)		
Brain Metastasis, N (%)			0.061	Chisq.test
No	701 (84.36%)	35 (4.21%)		
Yes	130 (15.64%)	1 (0.12%)		
Bone Metastasis, N (%)			0.959	Chisq.test
No	608 (73.16%)	27 (3.25%)		
Yes	223 (26.84%)	9 (1.08%)		
Adrenal Metastasis, N (%)			0.096	Fisher.test
No	745 (89.65%)	29 (3.49%)		
Yes	86 (10.35%)	7 (0.84%)		
Subcutaneous Metastasis, N (%)			0.098	Fisher.test
No	820 (98.68%)	34 (4.09%)		
Yes	11 (1.32%)	2 (0.24%)		
Muscle Metastasis, N (%)			0.473	Fisher.test
No	817 (98.32%)	35 (4.21%)		
Yes	14 (1.68%)	1 (0.12%)		
Meningeal Metastasis, N (%)			1.000	Fisher.test
No	830 (99.88%)	36 (4.33%)		
Yes	1 (0.12%)	0 (0)		
Pericardial Metastasis, N (%)			1.000	Fisher.test
No	811 (97.59%)	36 (4.33%)		
Yes	20 (2.41%)	0 (0)		
Intraabdominal Metastasis, N (%)			0.111	Fisher.test
No	729 (87.73%)	35 (4.21%)		
Yes	102 (12.27%)	1 (0.12%)		
Pancreatic Metastasis, N (%)			< 0.001	Fisher.test
No	813 (97.83%)	29 (3.49%)		
Yes	18 (2.17%)	7 (0.84%)		
Liver Metastasis, N (%)			0.128	Chisq.test
No	615 (74.01%)	22 (2.65%)		
Yes	216 (25.99%)	14 (1.68%)		
Non-draining Areas Metastasis, N (%)			< 0.001	Fisher.test
No	747 (89.89%)	22 (2.65%)		
Yes	84 (10.11%)	14 (1.68%)		
Pleural Metastasis, N (%)			0.243	Fisher.test
No	751 (90.37%)	35 (4.21%)		
Yes	80 (9.63%)	1 (0.12%)		
Age, Median (IQR)			0.303	Wilcoxon
Height, Median (IQR)			0.221	Wilcoxon
Weight, Median (IQR)			< 0.001	Wilcoxon
BMI, Median (IQR)			< 0.001	Wilcoxon
Cycles of Immunotherapy, Median (IQR)			< 0.001	Wilcoxon

*KPS* Karnofsky performance status, *BMI* body mass index, *IQR* interquartile range

**Fig. 3:**
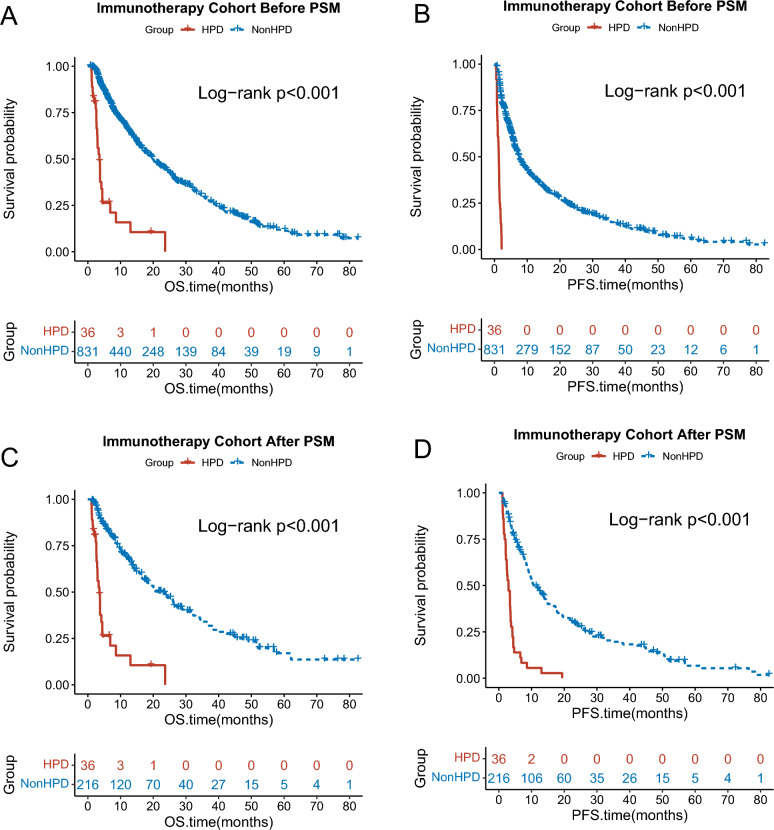
Survival analysis of subgroups. **A**. Kaplan-Meier curve for OS between HPD and non-HPD groups before PSM. **B**. Kaplan-Meier curve for PFS between HPD and non-HPD groups before PSM. **C**. Kaplan-Meier curve for OS between HPD and non-HPD groups after PSM. **D**. Kaplan-Meier curve for PFS between HPD and non-HPD groups after PSM

Survival analysis revealed that the OS and PFS of the HPD groups were significantly shorter than non-HPD group (OS: mOS: 3.57 [95%CI 2.86–4.40] months vs. 19.9 [95%CI 17.47–22.83] months, HR = 5.97 [95%CI 3.77–9.46], p < 0.001; PFS: mPFS: 1.32 [95%CI 1.00–1.50] months vs. 7.57 [95%CI 6.83–8.57] months, HR = 9.74 [95%CI 6.27–15.12], p < 0.001; Fig. 3A). To batch the intergroup imbalances on the clinical characteristics, PSM was performed. After PSM, clinical characteristics were listed in Table 2. The OS and PFS of the HPD group were still shorter than non-HPD group (OS: mOS: 3.57 [95%CI 2.87–4.40] months vs 19.90 [95% CI 17.47–22.83] months, HR = 6.09 [95% CI 2.40–15.45], p < 0.001; PFS: mPFS: 1.31 [95% CI 1.00–1.50] months vs 7.57 [95% CI 6.83–8.57] months, HR = 9.74 [95% CI 6.27–15.12], p < 0.001; Fig. 3B).

**Table 2:** Clinical characteristics of patients after PSM

Characteristics	Non-HPD	HPD	p value	Method
N	216	36		
Gender, N (%)			0.253	Chisq test
Female	58 (23.0%)	13 (5.2%)		
Male	158 (62.7%)	23 (9.1%)		
Pathology type, N (%)			0.112	Yates' correction
Adenocarcinoma	106 (42.1%)	22 (8.7%)		
Squamous	55 (21.8%)	12 (4.8%)		
Small Cell Carcinoma	29 (11.5%)	2 (0.8%)		
Sarcoma	2 (0.8%)	0 (0%)		
Others	24 (9.5%)	0 (0%)		
Differentiation, N (%)			0.150	Yates' correction
Low Differentiation	128 (50.8%)	18 (7.1%)		
Low To Median Differentiation	30 (11.9%)	3 (1.2%)		
Median Differentiation	34 (13.5%)	12 (4.8%)		
Median To High Differentiation	1 (0.4%)	0 (0)		
Unknown	23 (9.1%)	3 (1.2%)		
No gene mutation, N (%)			0.504	Chisq test
No	49 (19.4%)	10 (4.0%)		
Yes	167 (66.3%)	26 (10.3%)		
Ki67, N (%)			0.711	Yates' correction
0–25%	17 (6.7%)	3 (1.2%)		
26–50%	20 (7.9%)	5 (2%)		
51–75%	18 (7.1%)	1 (0.4%)		
76–100%	19 (7.5%)	4 (1.6%)		
Unknown	142 (56.3%)	23 (9.1%)		
TNM, N (%)			0.705	Yates' correction
III	20 (6.7%)	3 (1.2%)		
IV	194 (77.0%)	32 (12.7%)		
Unknown	2 (0.8%)	1 (0.4%)		
Smoking, N (%)			0.452	Yates' correction
Never	103 (40.9%)	20 (7.9%)		
Ever	76 (30.2%)	13 (5.2%)		
Current	33 (13.1%)	2 (0.8%)		
Unknown	4 (1.6%)	1 (0.4%)		
Drinking, N (%)			0.218	Yates' correction
Never	120 (47.6%)	22 (8.7%)		
Ever	37 (14.7%)	9 (3.6%)		
Current	56 (22.2%)	4 (1.6%)		
Unknown	3 (1.2%)	1 (0.4%)		
Family History of Tumor, N (%)			0.028	Yates' correction
Yes	61 (24.2%)	8 (3.2%)		
No	150 (59.5%)	24 (9.5%)		
Unknown	5 (2.0%)	4 (1.6%)		
KPS score, N (%)			0.948	Yates' correction
10–20 point	7 (2.8%)	1 (0.4%)		
30–40 point	2 (0.8%)	0 (0)		
70–80 point	46 (18.3%)	9 (3.6%)		
90–100 point	160 (63.5%)	26 (10.3%)		
Unknown	1 (0.4%)	0 (0)		
Combined Chemotherapy, N (%)			0.181	Chisq test
Yes	110 (43.7%)	14 (5.6%)		
No	106 (42.1%)	22 (8.7%)		
Combined Antiangiotherapeutics, N (%)			0.038	Chisq test
No	157 (62.3%)	32 (12.7%)		
Yes	59 (23.4%)	4 (1.6%)		
Combined Target Therapy,N (%)			1.000	Yates' correction
No	210 (83.3%)	35 (13.9%)		
Yes	6 (2.4%)	1 (0.4%)		
Combined Radiotherapy, N (%)			0.745	Yates' correction
No	197 (78.2%)	34 (13.5%)		
Yes	19 (7.5%)	2 (0.8%)		
Combined Other Therapy, N (%)			0.209	Chisq test
No	84 (33.3%)	18 (7.1%)		
Yes	132 (52.4%)	18 (7.1%)		
Lung Metastasis, N (%)			0.440	Chisq test
No	105 (41.7%)	20 (7.9%)		
Yes	111 (44.0%)	16 (6.3%)		
Brain Metastasis, N (%)			0.245	Chisq test
No	182 (72.2%)	33 (13.1%)		
Yes	34 (13.5%)	3 (1.2%)		
Bone Metastasis, N (%)			0.335	Chisq test
No	151 (59.9%)	28 (11.1%)		
Yes	65 (25.8%)	8 (3.2%)		
Adrenal Metastasis, N (%)			0.040	Yates' correction
No	187 (74.2%)	36 (14.3%)		
Yes	29 (11.5%)	0 (0)		
Subcutaneous Metastasis, N (%)			1.000	Yates' correction
No	213 (84.5%)	36 (14.3%)		
Yes	3 (1.2%)	0 (0)		
Muscle Metastasis, N (%)			0.598	Fisher test
No	210 (83.3%)	36 (14.3%)		
Yes	6 (2.4%)	0 (0)		
Meningeal Metastasis, N (%)			1.000	Fisher test
No	215 (85.3%)	36 (14.3%)		
Yes	1 (0.4%)	0 (0)		
Pericardial Metastasis, N (%)			0.598	Fisher test
No	210 (83.3%)	36 (14.3%)		
Yes	6 (2.4%)	0 (0)		
Intraabdominal Metastasis, N (%)			0.849	Chisq test
No	171 (67.9%)	29 (11.5%)		
Yes	45 (17.9%)	7 (2.8%)		
Pancreatic Metastasis, N (%)			0.782	Yates' correction
No	211 (83.7%)	36 (14.3%)		
Yes	5 (2.0%)	0 (0)		
Liver Metastasis, N (%)			0.169	Chisq test
No	143 (56.7%)	28 (11.1%)		
Yes	73 (29.0%)	8 (3.2%)		
Non-draining Areas Metastasis, N (%)			0.323	Yates' correction
No	209 (82.9%)	33 (13.1%)		
Yes	7 (2.8%)	3 (1.2%)		
Pleural Metastasis, N (%)			0.366	Yates' correction
No	197 (78.2%)	35 (13.9%)		
Yes	19 (7.5%)	1 (0.4%)		
Age, Median (IQR)	58 (51, 67)	58 (50.25, 66.25)	0.773	Wilcoxon
Height, Mean ± Sd	1.685 ± 0.074	1.668 ± 0.079	0.225	T test
Weight, Median (IQR)	63 (56, 71)	61 (51.75, 70)	0.309	Wilcoxon
BMI, Median (IQR)	22.519 (19.794, 24.612)	21.967 (18.423, 25.025)	0.755	Wilcoxon

*KPS* Karnofsky performance status, *BMI* body mass index, *IQR* interquartile range

**Fig. 4:**
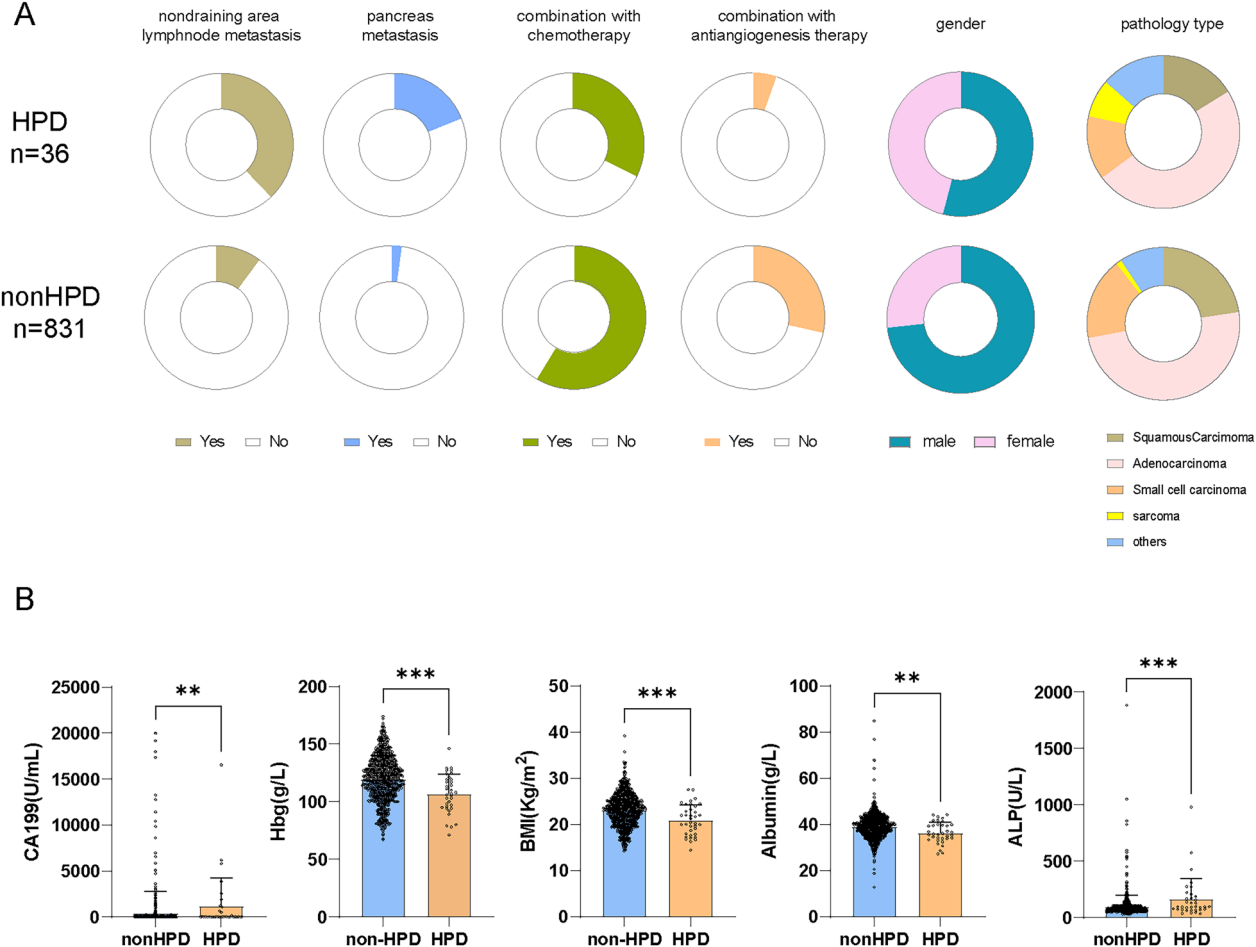
Differential analysis of clinical features between HPD and non-HPD groups. **A**. Differential analysis of clinical characteristics between HPD and non-HPD groups. **B**. Differential analysis of CA-199, Hbg, BMI, Albumin, and ALP between HPD and non-HPD groups (p<0.001 ***, p<0.01 **, p<0.05 *)

A comparative analysis of the clinical characteristics of the two groups were performed; there were significantly different in gender, pathology type, distant metastasis, and therapy regimen (Fig. 4A). The incidence of HPD group was significantly increased in female patients (HR = 2.20 [95% CI 1.12–4.31], p = 0.034), sarcoma pathology type (HR = 7.70 [95% CI 2.02–29.33], p < 0.001), non-draining area lymph node metastasis (HR = 5.66 [95% CI 2.79–11.48], p < 0.001), pancreatic metastasis (HR = 10.90 [95% CI 4.22–28.14], p < 0.001). However, ICI therapy combination with chemotherapy or anti-angiogenesis therapy could significantly decrease the incidence of HPD (Chemotherapy: HR = 0.52 [95%CI 0.32–0.85], p < 0.001; Anti-angiogenesis therapy: HR = 0.20 [95%CI: 0.05–0. 75], p < 0.001).

In our cohorts, we found lab tests including CA-199 (non-HPD vs HPD: 450.19 ± 2327.08 U/L vs 1173.55 ± 3098.89 U/L, p = 0.004), hemoglobin (Hbg) (119.19 ± 21.35 g/L vs 106.94 ± 16.95 g/L, p < 0.001), albumin(ALB) (39.09 ± 5.40 g/L vs 36.47 ± 4.53 g/L, p = 0.005), and alkaline phosphatase (ALP) (97.22 ± 100.78 U/L vs 166.45 ± 182.40 U/L, p = 0.032) were significantly different between HPD and non-HPD patients (Fig. 4B). The age showed no significantly difference in two groups (Additional file 1: Fig. S1A). Other lab tests, including white blood cell (WBC), neutrophil (NEU) to WBC ratio, lymphocyte (LYM) to WBC ratio, neutrophil to lymphocyte ratio (NLR), platelet (PLT), platelet to lymphocyte ratio (PLR), lactate dehydrogenase (LDH) distributed equally in two groups. (Additional file 1: Fig. S1B–H).

Based on the risky factors participated in our study, subgroup analysis performed by separate tumor types. Cholangiocarcinoma had the highest HPD incidence (7/42, 16.7%), followed by colon cancer (3/25, 12.0%) and esophageal carcinoma (3/26, 11.5%). The subgroup analysis was shown in Additional file 3: Table S1. In cholangiocarcinoma, pancreatic metastasis (p = 0.023), distant lymph node metastasis (p = 0.044), ALB (p = 0.028) and Hbg (p = 0.003) was relevant to the high incidence of HPD. In colorectal cancer, lower BMI (p = 0.009) was found in HPD patients. In lung adenocarcinoma (LUAD), the distribution of pancreatic metastasis (p < 0.001), non-draining area lymph node metastasis (p = 0.042), and ALP (p = 0.021) was significantly different in HPD and non-HPD groups. Besides, risk factors were compared based on gender in HPD patients. There is no statistical difference in two subgroups (Additional file 4: Table S2).

**Fig. 5:**
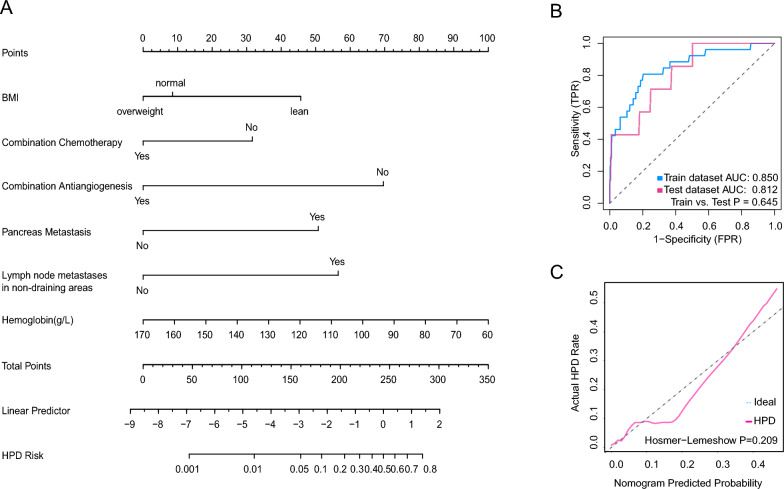
Construction of predictive nomogram model. **A**. The nomogram model for predicting the occurrence of HPD. **B**. ROC curve for train and test dataset. **C**. Hosmer-Lemeshow test indicates that the nomogram model fits well between predictive HPD incidence and actual HPD incidence

Through multivariable analysis and model training, six risk factors, including BMI, Hbg, whether received combination chemotherapy, received combination anti-angiogenesis therapy, whether existing pancreatic metastasis, and whether existing distant lymph node metastasis were independent factors to predict HPD occurrence. We visualized the risk factors by nomogram models. Briefly, patients with lower BMI, lower hemoglobin, existing pancreas or lymph node distant metastasis and received anti-PD-1 monotherapy were more inclined to occur HPD after receiving immunotherapy (Fig. 5A). This model had an excellent predicting ability. The AUC of the training dataset was 0.850. However, the AUC of the test dataset was 0.812 (Fig. 5B). The precision recall curve analysis showed average precision was 0.757 in train dataset and 0.656 in the test dataset (Additional file 2: Fig. S2). Additionally, the Hosmer–Lemeshow test revealed that the fit between nomogram predicted probability and actual HPD rate was particularly good (Fig. 5C).

## Discussion

HPD is a novel concept arising from the clinical application of ICI drugs in malignant patients and remains one of the challenges of cancer immunotherapy due to its poor prognosis [1]. Exploring the critical molecular mechanisms, screening the typical clinical and experimental signatures, and establishing precise predictive models are of great importance in current IO research. The major finding in this study is identifying typical clinical signatures of the HPD population among pan-cancer patients who received ICI therapy and constructing a feasible predictive model based on clinical characteristics and lab tests.

In previous studies, the definition of HPD after ICIs treatment varies in different measurements of poor prognosis, including the growth rate of target lesions, the number of new lesions and the time from ICI administration to treatment failure [7]. Due to no consensus on the optimal definition, accurately screening typical HPD groups is complex and may cause heterogeneity in real-world studies. Therefore, it is crucial to choose an appropriate criterion method. Three criteria are commonly used to define HPD patients in previous reports: tumor growth kinetics (TGK), tumor growth rate (TGR), and time to treatment failure (TTF). TTF is relatively easy to calculate but is affected not only by the deterioration of malignancy but also the adverse events and the subjective intention of patients. In contrast, TGK and TGR require the assessment of tumor burden at different time points according to RECIST 1.1 criteria and are more objective in reflecting the progression of tumor lesions. Kim et al. compared these three measurements in non small cell lung cancer (NSCLC) patients and reported that the concordance rate of each criterion was higher between TGK and TGR, indicating that the definition based on the tumor growth dynamics may be more accurate and may be used more universally than the definition based on TTF. In this study, TGK was chosen to evaluate the change in tumor burden after ICI treatment, and we screened the patients from more than 3,000 immunotherapy cases TGK was calculated for each patient with completely imagined messages around ICI therapy. According to previous reports, The HPD cases were strictly defined as TGK > 2. Although the incidence of HPD in this study is 4.3%, lower than in previous literature, the more typically HPD groups are identified [5, 23].

TGKr can reflect changes in tumor growth kinetics, which is considered the appropriate method to evaluate HPD [17]. However, it requires pre-baseline and baseline imaging assessment, which is difficult to achieve in first-line patients. In our study, we strictly screened the patients with completely imagine messages around ICI therapy from more than 3000 immunotherapy cases and used TGKr to assess HPD groups. Consequently, although the incidence of HPD, about 4.3%, in our cohort was less than in previous literature, the more typically HPD groups were identified. Notably, we found the relatively higher proportion with HPD occurence, approximately 16.7%, in cholangiocarcimnoma patients after reciving ICIs. Furthermore, patients with pancreatic metastasis or distant lymph node metastasis had more tendency to develop HPD. The phenomenon appeared in other tumor types, especially in LUAD, which seldomly coexsited with pancreatic metastasis.

After clinical correlation studies, we found that females, sarcoma, low BMI, distant metastasis type, and whether combined with anti-angiogenic therapy were signature predictors to distinguish HPD groups. Lab tests, including CA-199, Hbg, ALB, and ALP, were closely connected with HPD.Hbg, BMI, and ALB showed body nutrition conditions in different aspects. This is in accordance with sarcopenic patients having a higher risk of progression in antiPD-1/PD-L1 agents treatment [24]. Sarcoma is a pathological type with a higher heterogeneous in vascular proliferations. In this study, combination with antiangiogenesis therapy could decrease the incidence of HPD. Thus, the poor nutritional status and abnormal angiogenesis may be potential mechanisms of HPD. To provide a convenient assessment method for clinical use, we constructed a nomogram model by independent factors for HPD prediction. The AUC of HPD models was 0.850 in the training group and 0.812 in the test dataset. It is regarded as a pretty accurate prediction ability and accessible indicators, which are immediate clinical values for a treatment plan.

Our study has advantages compared to established clinical predictive models for HPD. First, more patients who received ICI therapy were included in our cohort; complete imaging information, appropriate assessment, and sufficient follow-up ensured that HPD patients were strictly distinguished from natural progression, adverse events, and pseudo-progression disease. Compared with an imaging approach-based prediction model [25], a clinical characteristic model is more feasible in the clinic. In addition, our model involves fewer indicators but more vital prediction ability, suitable for clinical use. Current studies for HPD are almost mono-cancer research [26–28]. Our study enrolled more than 17 types of solid tumors as the first pan-cancer clinical research, reflecting the characteristic of HPD, which appears in all tumor types [17].

The mechanism underlying HPD was ambiguous and complex. Single gene mutation or signaling pathway regulation cannot fully explain the immune cell dysfunction and changes in the tumor microenvironment. However, the clinical phenomena of HPD will provide hints for basic research. Our study found that patients with anemia seem to benefit less from immunotherapy and have the propensity to develop HPD.

Anemia has been confirmed to be associated with various malignant tumors and reported as a risk factor for prognostic prediction [29]. Recently research focused on the connection between cancer-related anemia and ICI therapy efficacy. Anemia patients are usually associated with poor outcomes after immunotherapy. However, the phenomenon still needs more rigorous theoretical justification. Zhu et al. study has pointed out that malignant tumors will induce anemia and initiate extramedullary hematopoiesis, which results in abnormal CD45^+^ endothelial progenitor cells (EPCs) accumulated in the spleen and liver. Consequently, this subgroup of CD45^+^ EPCs will differentiate into erythroid differentiated myeloid cells, a tumor-associated myeloid cell population, cause a suppressive microenvironment and impair the efficacy of immunotherapy [30]. Nevertheless, there is still no report revealing the mechanism underlying anemia and HPD, which may become a novel point to explain the incidence of HPD.

Our study has limitations. As a retrospective study, confounding factors cannot be avoided. Classical biomarkers for evaluating ICI efficacy, such as PD-L1 expression levels and tumor mutation burden [18], might provide more cues for HPD diagnosis. Unfortunately, this part of the data needs to be completed. In addition, limited by the actual situation, routine genetic testing is not recommended. Aberrant activation of the cancer pathway mediated by gene mutation is an essential loop of the pathogenic mechanism. Our follow-up work will focus on the mechanism behind it. Finally, the HPD model still needs validation in external cohorts.

In conclusion, we conducted pan-cancer research to solve the unmet need for a predictive method of HPD. Through rigorous screening and analysis, we constructed a clinically feasible and practical model for predicting HPD incidents, which could help oncologists to stratify risky patients and select treatment strategies. Moreover, we put forward clinical evidence that anemia is closely connected with HPD, providing a novel point for future studies.

### Supplementary Information

**Additional file 1: Figure S1**. Differential analysis of no statistically significant continuous variables between HPD and non-HPD groups (ns, no significance). Abbr: WBC, white blood cell; NEU/WBC ratio, neutrophil to white blood cell ratio; LYM/WBC ratio, lymphocyte to white blood cell ratio; NLR, neutrophil to lymphocyte ratio; PLT, platelet; PLR, platelet to lymphocyte ratio; LDH, lactate dehydrogenase.

**Additional file 2: Figure S2**. The precision-recall curve of nomogram model in HPD prediction.

**Additional file 3: Table S1**. Subgroup analysis of risk factors based on tumor types.

**Additional file 4: Table S2**. Subgroup analysis of risk factors in HPD groups.

## Data Availability

Raw data were generated at The Fifth Medical Center of PLA General Hospital, Chinese PLA General Hospital. Derived data supporting the findings of this study are available from the corresponding author on request.
