# Supplementary figures and images for: Prediction model for hyperprogressive disease in patients with advanced solid tumors received immune-checkpoint inhibitors: a pan-cancer study
Source: Cancer Cell Int. 2023 Sep 30;23:224. doi: 10.1186/s12935-023-03070-x (PMC10543870; doi:10.1186/s12935-023-03070-x)

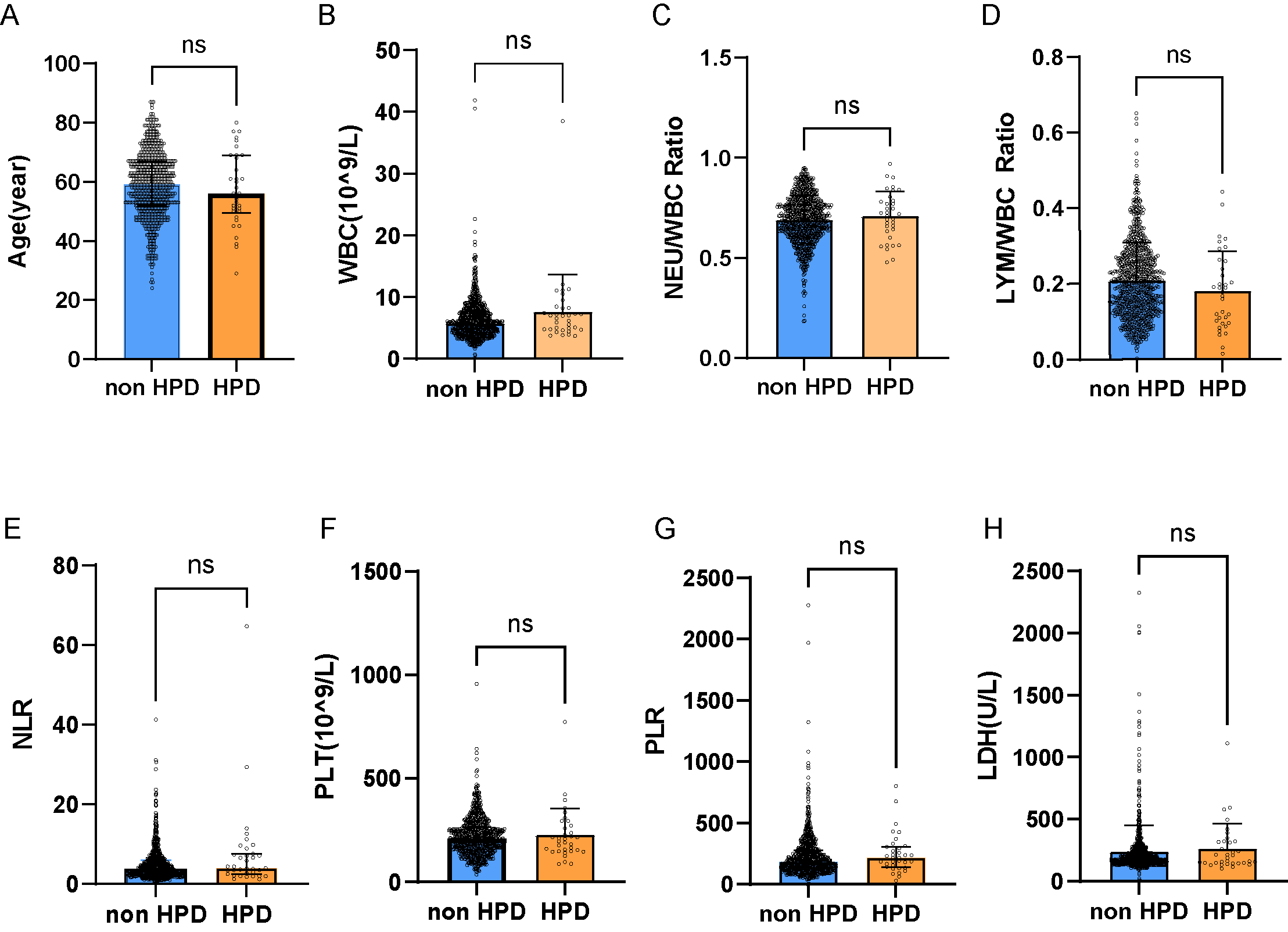

Supplement: Supplementary file 1 — Additional file 1: Figure S1. Differential analysis of no statistically significant continuous variables between HPD and non-HPD groups (ns, no significance). Abbr: WBC, white blood cell; NEU/WBC ratio, neutrophil to white blood cell ratio; LYM/WBC ratio, lymphocyte to white blood cell ratio; NLR, neutrophil to lymphocyte ratio; PLT, platelet; PLR, platelet to lymphocyte ratio; LDH, lactate dehydrogenase. [file 12935_2023_3070_MOESM1_ESM.tif]

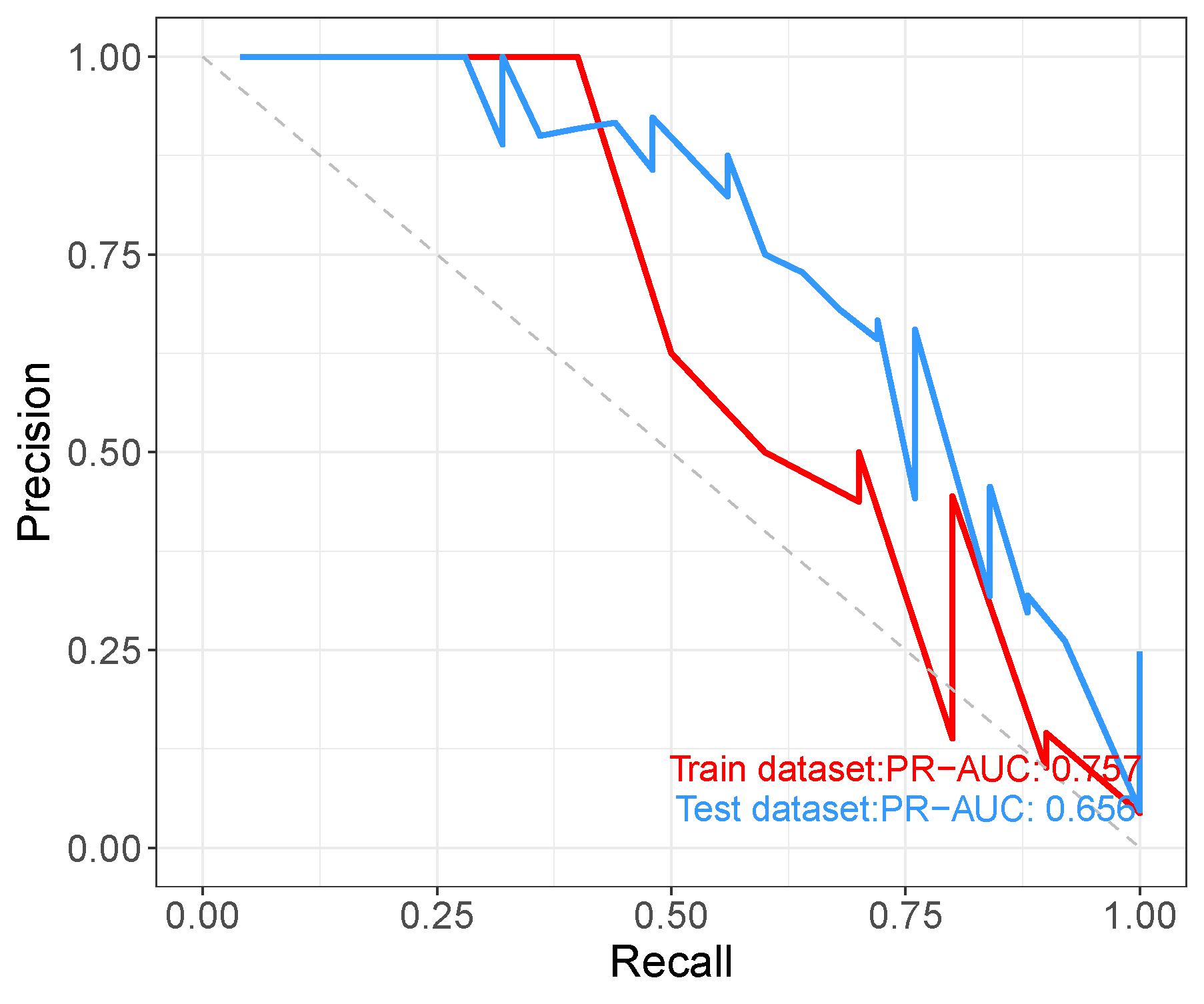

Supplement: Supplementary file 2 — Additional file 2: Figure S2. The precision-recall curve of nomogram model in HPD prediction. [file 12935_2023_3070_MOESM2_ESM.tif]
