# Supplementary material for: Prediction model for hyperprogressive disease in patients with advanced solid tumors received immune-checkpoint inhibitors: a pan-cancer study
Source: Cancer Cell Int. 2023 Sep 30;23:224. doi: 10.1186/s12935-023-03070-x (PMC10543870; doi:10.1186/s12935-023-03070-x)
Supplement: Supplementary file 3 — Additional file 3: Table S1. Subgroup analysis of risk factors based on tumor types. [file 12935_2023_3070_MOESM3_ESM.docx]

　　 Supplementary Table 1. Subgroup analysis of risk factors based on tumor types.

| Characteristics | HPD | Non-HPD | p value | Method |
| --- | --- | --- | --- | --- |
| Cholangiocarcimnoma |  |  |  |  |
| N | 7 | 35 |  |  |
| Gender |  |  | 1.000 | Yates' correction |
| female | 2 | 7 |  |  |
| male | 5 | 28 |  |  |
| Combined chemotherapy |  |  | 0.313 | Yates' correction |
| Yes | 4 | 29 |  |  |
| No | 3 | 6 |  |  |
| Combined antiangiotherapy |  |  | 0.814 | Yates' correction |
| No | 7 | 31 |  |  |
| Yes | 0 | 4 |  |  |
| **Pancreatic metastasis** |  |  | **0.023** | **Yates' correction** |
| No | 5 | 35 |  |  |
| Yes | 2 | 0 |  |  |
| **Non-draining area lymph node metastasis** |  |  | **0.044** | **Yates' correction** |
| No | 3 | 30 |  |  |
| Yes | 4 | 5 |  |  |
| CA-199, median (IQR) | 1238 (275.42 - 3224.5) | 116.3 (27.29 - 481.05) | 0.177 | Wilcoxon |
| **Hemoglobin, mean ± SD** | **99.714 ± 8.2404** | **114.4 ± 18.857** | **0.003** | **Welch t' test** |
| **Albumin, mean ± SD** | **33.357 ± 4.4109** | **38.437 ± 5.5524** | **0.028** | **T test** |
| ALP, median (IQR) | 171.9 (114.55 - 266.5) | 124.5 (81 - 179.85) | 0.303 | Wilcoxon |
| BMI, mean ± SD | 19.236 ± 2.9865 | 21.754 ± 3.0431 | 0.052 | T test |
|  |  |  |  |  |
| Colorectal cancer |  |  |  |  |
| N | 3 | 22 |  |  |
| Gender |  |  | 1.000 | Fisher test |
| male | 2 | 16 |  |  |
| female | 1 | 6 |  |  |
| Combined chemotherapy |  |  | 1.000 | Fisher test |
| No | 2 | 11 |  |  |
| Yes | 1 | 11 |  |  |
| Combined antiangiotherapy |  |  | 0.534 | Fisher test |
| No | 3 | 15 |  |  |
| Yes | 0 | 7 |  |  |
| Pancreatic metastasis |  |  | 0.120 | Fisher test |
| No | 2 | 22 |  |  |
| Yes | 1 | 0 |  |  |
| Non-draining area lymph node metastasis |  |  | 0.180 | Fisher test |
| No | 1 | 17 |  |  |
| Yes | 2 | 5 |  |  |
| CA-199, mean ± SD | 641.57 ± 461.07 | 999.74 ± 1931.6 | 0.756 | T test |
| Hbg, mean ± SD | 110 ± 16.371 | 117.41 ± 21.311 | 0.571 | T test |
| Albumin, mean ± SD | 36.067 ± 7.7655 | 37.991 ± 3.6605 | 0.462 | T test |
| ALP, mean ± SD | 195.13 ± 201.28 | 92.573 ± 41.26 | 0.471 | Welch t' test |
| **BMI, mean ± SD** | **17.087 ± 0.31902** | **22.788 ± 3.411** | **0.009** | **T test** |
|  |  |  |  |  |
| Esophageal carcinoma |  |  |  |  |
| N | 3 | 23 |  |  |
| Gender |  |  | 1.000 | Fisher test |
| male | 2 | 18 |  |  |
| female | 1 | 5 |  |  |
| Combined chemotherapy |  |  | 0.556 | Fisher test |
| No | 2 | 9 |  |  |
| Yes | 1 | 14 |  |  |
| Combined antiangiotherapy |  |  | 1.000 | Fisher test |
| No | 3 | 19 |  |  |
| Yes | 0 | 4 |  |  |
| Non-draining area lymph node metastasis |  |  | 0.488 | Fisher test |
| No | 2 | 19 |  |  |
| Yes | 1 | 4 |  |  |
| CA-199, mean ± SD | 22.15 ± 4.879 | 36.171 ± 53.635 |  |  |
| Hbg, mean ± SD | 120 ± 9.8995 | 117 ± 16.393 |  |  |
| Albumin, mean ± SD | 37.6 ± 5.2326 | 40.952 ± 6.3625 |  |  |
| ALP, mean ± SD | 63.2 ± 23.476 | 92.217 ± 54.517 |  |  |
| BMI, mean ± SD | 20.313 ± 2.0737 | 21.371 ± 3.3876 | 0.606 | T test |
|  |  |  |  |  |
| LUSC |  |  |  |  |
| N | 6 | 152 |  |  |
| Gender |  |  | 0.118 | Yates' correction |
| female | 4 | 43 |  |  |
| male | 2 | 109 |  |  |
| Combined chemotherapy |  |  | 0.179 | Yates' correction |
| No | 5 | 71 |  |  |
| Yes | 1 | 81 |  |  |
| Combined antiangiotherapy |  |  | 0.594 | Fisher test |
| No | 6 | 129 |  |  |
| Yes | 0 | 23 |  |  |
| Non-draining area lymph node metastasis |  |  | 0.301 | Fisher test |
| No | 5 | 144 |  |  |
| Yes | 1 | 8 |  |  |
| CA-199, median (IQR) | 29.435 (14.808 - 57.953) | 11.04 (6.7525 - 18.75) | 0.053 | Wilcoxon |
| Hbg, median (IQR) | 111 (103.5 - 126) | 125 (109.5 - 136) | 0.329 | Wilcoxon |
| Albumin, median (IQR) | 34.7 (33.575 - 38.3) | 38.6 (35.625 - 41.275) | 0.210 | Wilcoxon |
| ALP, median (IQR) | 80.3 (47.925 - 97.45) | 76.5 (64.675 - 93.6) | 0.797 | Wilcoxon |
| BMI, mean ± SD | 20.625 ± 4.5436 | 23.322 ± 3.533 | 0.071 | T test |
|  |  |  |  |  |
| LUAD |  |  |  |  |
| N | 4 | 244 |  |  |
| Gender |  |  | 1.000 | Yates' correction |
| male | 3 | 173 |  |  |
| female | 1 | 71 |  |  |
| Combined chemotherapy |  |  | 0.472 | Yates' correction |
| No | 3 | 108 |  |  |
| Yes | 1 | 136 |  |  |
| Combined antiangiotherapy |  |  | 0.247 | Yates' correction |
| No | 4 | 143 |  |  |
| Yes | 0 | 101 |  |  |
| **Pancreatic metastasis** |  |  | **p<0.001** | **Yates' correction** |
| No | 2 | 240 |  |  |
| Yes | 2 | 4 |  |  |
| **Non-draining area lymph node metastasis** |  |  | **0.042** | **Yates' correction** |
| No | 2 | 224 |  |  |
| Yes | 2 | 20 |  |  |
| CA-199, median (IQR) | 15.63 (11.627 - 21.083) | 18.55 (8.685 - 50.11) | 0.545 | Wilcoxon |
| Hbg, median (IQR) | 115.5 (107 - 122.5) | 122 (107 - 134) | 0.381 | Wilcoxon |
| Albumin, median (IQR) | 36.15 (35.25 - 38.125) | 38.85 (35.9 - 42.325) | 0.337 | Wilcoxon |
| **ALP, median (IQR)** | **141.8 (95.15 - 209.6)** | **76.05 (62.925 - 98.3)** | **0.021** | **Wilcoxon** |
| BMI, mean ± SD | 22.677 ± 2.3715 | 23.279 ± 3.2775 | 0.715 | T test |
|  |  |  |  |  |
| SCLC |  |  |  |  |
| N | 3 | 136 |  |  |
| Gender |  |  | 0.569 | Yates' correction |
| male | 1 | 90 |  |  |
| female | 2 | 46 |  |  |
| Combined chemotherapy |  |  | 0.192 | Yates' correction |
| Yes | 1 | 110 |  |  |
| No | 2 | 26 |  |  |
| Combined antiangiotherapy |  |  | 0.903 | Yates' correction |
| No | 3 | 109 |  |  |
| Yes | 0 | 27 |  |  |
| Pancreatic metastasis |  |  | 1.000 | Yates' correction |
| No | 3 | 132 |  |  |
| Yes | 0 | 4 |  |  |
| **Non-draining area lymph node metastasis** |  |  | **0.002** | **Yates' correction** |
| No | 1 | 129 |  |  |
| Yes | 2 | 7 |  |  |
| CA-199, mean ± SD | 16.52 ± 13.787 | 51.411 ± 286.15 | 0.834 | T test |
| Hbg, mean ± SD | 117.33 ± 8.6217 | 123.03 ± 20.802 | 0.638 | T test |
| Albumin, mean ± SD | 38.833 ± 1.5308 | 39.681 ± 4.5123 | 0.747 | T test |
| ALP, mean ± SD | 104.17 ± 55.42 | 90.945 ± 81.606 | 0.781 | T test |
| BMI, mean ± SD | 22.323 ± 1.9394 | 24.705 ± 3.5525 | 0.250 | T test |
|  |  |  |  |  |
| Urinary |  |  |  |  |
| N | 2 | 20 |  |  |
| Gender |  |  | 0.338 | Fisher test |
| female | 1 | 3 |  |  |
| male | 1 | 17 |  |  |
| Combined chemotherapy |  |  | 1.000 | Fisher test |
| No | 1 | 12 |  |  |
| Yes | 1 | 8 |  |  |
| Combined antiangiotherapy |  |  | 0.481 | Fisher test |
| No | 1 | 15 |  |  |
| Yes | 1 | 5 |  |  |
| Non-draining area lymph node metastasis |  |  | 1.000 | Fisher test |
| No | 2 | 14 |  |  |
| Yes | 0 | 6 |  |  |
| CA-199, mean ± SD | 10.15 ± 1.9658 | 100.04 ± 170.11 |  |  |
| Hbg, mean ± SD | 89.5 ± 14.849 | 114.63 ± 16.402 |  |  |
| Albumin, mean ± SD | 39.75 ± 0.21213 | 40.921 ± 4.165 |  |  |
| ALP, mean ± SD | 180.05 ± 130.46 | 70.988 ± 18.825 |  |  |
| BMI, mean ± SD | 22.382 ± 2.829 | 24.989 ± 3.1942 |  |  |
|  |  |  |  |  |
| Others |  |  |  |  |
| N | 4 | 60 |  |  |
| Gender |  |  | 0.926 | Yates' correction |
| male | 4 | 51 |  |  |
| female | 0 | 9 |  |  |
| Combined chemotherapy |  |  | 0.121 | Yates' correction |
| No | 4 | 28 |  |  |
| Yes | 0 | 32 |  |  |
| Combined antiangiotherapy |  |  | 0.795 | Yates' correction |
| No | 3 | 33 |  |  |
| Yes | 1 | 27 |  |  |
| Pancreatic metastasis |  |  | 0.069 | Yates' correction |
| No | 3 | 60 |  |  |
| Yes | 1 | 0 |  |  |
| Non-draining area lymph node metastasis |  |  | 1.000 | Yates' correction |
| No | 3 | 53 |  |  |
| Yes | 1 | 7 |  |  |
| CA-199, median (IQR) | 18.71 (12.763 - 1467.9) | 12.62 (7.325 - 25.325) | 0.442 | Wilcoxon |
| Hbg, mean ± SD | 97.5 ± 23.868 | 113.84 ± 21.162 | 0.143 | T test |
| Albumin, mean ± SD | 35.55 ± 6.0693 | 37.996 ± 4.5449 | 0.312 | T test |
| **ALP, median (IQR)** | **169.25 (122.92 - 400.32)** | **70.3 (55.9 - 102.98)** | **0.018** | **Wilcoxon** |
| BMI, mean ± SD | 21.463 ± 4.2704 | 21.954 ± 3.4074 | 0.784 | T test |
| Abbr: Hbg, hemoglobin; ALP, alkaline phosphatase; BMI, body mass index; LUSC, lung squamous cell carcinoma; LUAD, lung adenocarcinoma; IQR, interquartile range; SD, Standard Deviation. | | | | |
