# Supplementary material for: Prediction model for hyperprogressive disease in patients with advanced solid tumors received immune-checkpoint inhibitors: a pan-cancer study
Source: Cancer Cell Int. 2023 Sep 30;23:224. doi: 10.1186/s12935-023-03070-x (PMC10543870; doi:10.1186/s12935-023-03070-x)
Supplement: Supplementary file 4 — Additional file 4: Table S2. Subgroup analysis of risk factors in HPD groups. [file 12935_2023_3070_MOESM4_ESM.docx]

Supplementary Table 2. Subgroup analysis of risk factors in HPD groups.

| Characteristics | Female | Male | p value | Method |
| --- | --- | --- | --- | --- |
| N | 13 | 23 |  |  |
| Combined chemotherapy |  |  | 0.708 | Fisher test |
| Yes | 3 | 8 |  |  |
| No | 10 | 15 |  |  |
| Combined antiangiotherapy |  |  | 0.525 | Fisher test |
| No | 13 | 21 |  |  |
| Yes | 0 | 2 |  |  |
| Pancreatic metastasis |  |  | 0.382 | Fisher test |
| No | 12 | 17 |  |  |
| Yes | 1 | 6 |  |  |
| Non-draining area lymph node metastasis |  |  | 0.723 | Fisher test |
| No | 7 | 15 |  |  |
| Yes | 6 | 8 |  |  |
| CA-199, median (IQR) | 24.53 (11.87 - 281.36) | 25.6 (15.585 - 942.4) | 0.537 | Wilcoxon |
| Hbg, mean ± SD | 100.27 ± 15.869 | 109.61 ± 17.122 | 0.138 | T test |
| Albumin, mean ± SD | 35.2 ± 3.494 | 36.739 ± 4.7474 | 0.347 | T test |
| ALP, median (IQR) | 168.1 (86.15 - 247.65) | 93.2 (70.35 - 135.25) | 0.153 | Wilcoxon |
| BMI, mean ± SD | 20.88 ± 2.4793 | 20.813 ± 3.6974 | 0.954 | T test |
| Abbr: Hbg, hemoglobin; ALP, alkaline phosphatase; BMI, body mass index; IQR, interquartile range; SD, Standard Deviation. | | | | |
